# Synthetic Pre-miRNA-Based shRNA as Potent RNAi Triggers

**DOI:** 10.4061/2011/131579

**Published:** 2011-07-12

**Authors:** Kazuya Terasawa, Kazuharu Shimizu, Gozoh Tsujimoto

**Affiliations:** ^1^Department of Pharmacogenomics, Graduate School of Pharmaceutical Sciences, Kyoto University, 46-29 Yoshida Shimoadachi-cho, Sakyo-ku, Kyoto 606-8501, Japan; ^2^Department of Nanobio Drug Discovery, Graduate School of Pharmaceutical Sciences, Kyoto University, 46-29 Yoshida Shimoadachi-cho, Sakyo-ku, Kyoto 606-8501, Japan

## Abstract

RNA interference (RNAi) is a powerful tool for studying gene function owing to the ease with which it can selectively silence genes of interest, and it has also attracted attention because of its potential for therapeutic applications. Chemically synthesized small interfering RNAs (siRNAs) and DNA vector-based short hairpin RNAs (shRNAs) are now widely used as RNAi triggers. In contrast to expressed shRNAs, the use of synthetic shRNAs is limited. Here we designed shRNAs modeled on a precursor microRNA (pre-miRNA) and evaluated their biological activity. We demonstrated that chemically synthetic pre-miRNA-based shRNAs have more potent RNAi activity than their corresponding siRNAs and found that their antisense strands are more efficiently incorporated into the RNA-induced silencing complex. Although greater off-target effects and interferon responses were induced by shRNAs than by their corresponding siRNAs, these effects could be overcome by simply using a lower concentration or by optimizing and chemically modifying shRNAs similar to synthetic siRNAs. These are challenges for the future.

## 1. Introduction

RNA interference (RNAi) is an evolutionarily conserved, gene-silencing mechanism that is triggered by double-stranded RNA (dsRNA). Two types of small RNA—namely, small interfering RNA (siRNA) and microRNA (miRNA)—are central players in RNAi. Both siRNAs and miRNAs regulate gene expression by annealing to mRNA sequence elements that are fully or partially complementary [1, 2]. Since transfected synthetic siRNAs were shown to induce RNAi in mammalian cells [3], they have been widely used to decipher gene function through suppression of gene expression, and they have also attracted attention because of their potential for therapeutic applications [4, 5]. miRNAs are a phylogenetically conserved family of endogenous small RNAs that play important roles in a wide variety of biological functions, including cell differentiation, tumor genesis, apoptosis, and metabolism [1, 2, 6, 7]. 

 miRNAs are initially generated as long primary transcripts (pri-miRNA) that are processed in the nucleus by the enzyme complexes Drosha and DiGeorge Critical Region 8 (DGCR8) to a 70–90 nt stem-loop structure called pre-miRNA. The pre-miRNA is then exported to the cytoplasm. There, the exported pre-miRNA or exogenous dsRNA is cleaved by the enzyme Dicer into a ~22-nucleotide (nt) duplex known as miRNA or siRNA, respectively. The duplex is then incorporated into the RNA-induced silencing complex (RISC). After removing one strand called the passenger strand, the remaining strand, called the guide strand, in the RISC guides the silencing complex to target mRNAs. Thus, downstream of their initial processing, siRNAs and miRNAs share the same cellular machinery [1, 2].

 Understanding the mechanism of the RNAi pathway has led to the development of alternative approaches of RNAi. Several groups have developed artificial miRNAs in the form of short-hairpin structures, called shRNAs, instead of siRNAs [8, 9]. DNA vector-based shRNAs are widely used. By contrast, the use of synthetic shRNAs is very limited [10–12], although synthetic shRNAs can easily incorporate chemical modifications to improve their stability and biological activity, similar to synthetic siRNAs. Probably, this limited use is because the chemical synthesis of long RNA oligonucleotides is generally difficult, consequently leading to high cost and low yield. Unlike DNA oligonucleotides, synthesis of RNA is less efficient owing to problems caused by the presence of the 2′-hydroxyl group of ribose, which requires selective protection during oligonucleotide assembly. Recently, a new protecting group, 2-cyanoethoxymetyhyl (CEM), has been developed [13]. By improving capping and coupling conditions, the authors succeeded in synthesizing a 110 mer pre-miRNA candidate and confirmed its biological activity [14]. Thus, synthetic long oligonucleotides are becoming a reality at a reasonable cost. In this study, we have designed shRNAs modeled on pri-miRNA and have evaluated their biological activity.

## 2. Materials and Methods

### 2.1. Synthetic RNA Oligonucleotides

**Figure 1:**
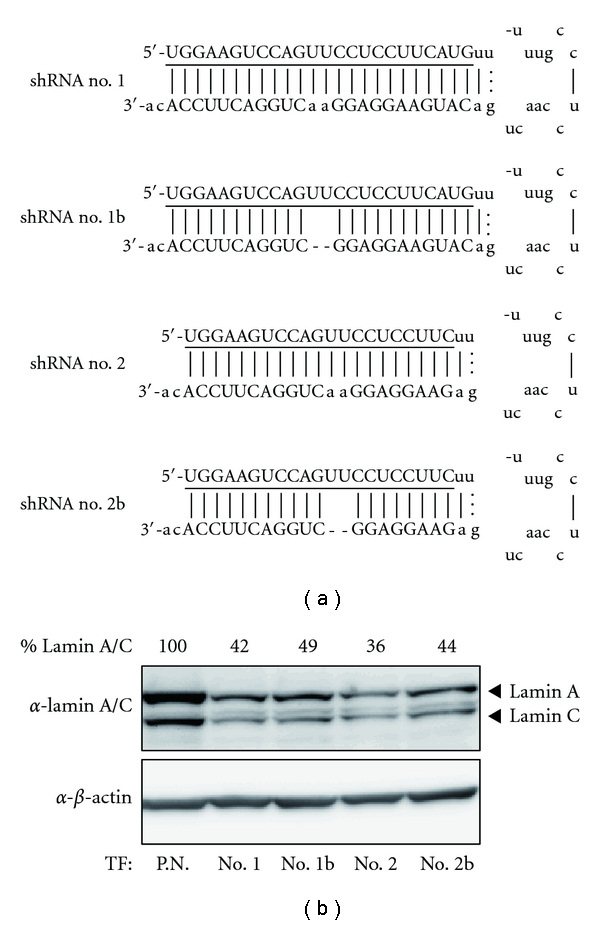
Design of pre-miRNA-based shRNAs and their RNAi activity. (a) The set of four synthetic shRNAs containing a 21 or 24 bp stem, either with or without a 2 bp deletion of the 3′ arm, is represented. The underlined sequences are complementary to the LMNA mRNA sequence. The lower cases are derived from the human pre-miR-155 sequence. (b) The RNAi activity of these shRNAs targeting LMNA was analyzed by immunoblotting. TF is the abbreviation for transfection.

The siRNA sequence targeting LMNA (GenBank accession number: NM_170707) was from position 600–620 relative to the start codon. The shRNAs in Figure 1(a) were kindly provided by Nippon Shinykau Co., Ltd. (Kyoto, Japan). These RNA oligonucleotides were synthesized as previously described [14]. To anneal shRNAs, shRNAs suspended in water were incubated for 5 min at 95°C in a heat block and then left until the block reached 25°C according to the manufacturer's protocol. Control (siGENOME Non-Targeting siRNA no. 2, denoted as “ctrl”) and custom designed siRNAs for LMNA were purchased from Dharmacon Research (Lafayette, CO, USA). Negative control pre-miRNA (denoted “P.N.”) was from Ambion (Austin, TX, USA).

### 2.2. Plasmid Construction

Human Ago2 (hAgo2) cDNA was amplified by PCR using the primers 5′-GGATCCATGTACTCGGGAGCCGGC-3′ and 5′-GCGGCCGCT CAAGCAAAGTACATGGTG-3′ after reverse transcription from total RNA isolated from HeLa cells and cloned into the pCR-blunt vector (Invitrogen, Carlsbad, CA, USA). A *Kpn*I and *Not*I fragment containing a FLAG-tag coding region in the pcDNA3FLAG vector (kindly gift from E. Nishida, Kyoto University, Japan) was ligated with the *Kpn*I and *Not*I-digested expression vector pcDNA5FRT (Invitrogen), yielding the pcDNA5FLAG vector. A *BamH*I and *Not*I fragment of hAgo2 cDNA in the pCR-Blunt vector was ligated with *BamH*I and *Not*I-digested pcDNA5FLAG vector, yielding the pcDNA5FLAG–hAgo2 plasmid. An *Eco*RI site was introduced into the *Xba*I site of the luciferase reporter vector pGL4.13 (Promega, Madison, WI, USA) by ligation with the oligonucleotides 5′-CTAGACTGAATTC-3′ and 5′-CTAGGAATTCAGT-3′, yielding the pGL4.13EcoRI vector. The oligonucleotides 5′-CTAGAGAAGGAGGAACTGGACTTCCAG-3′ and 5′-AATTCTGGAAGTCCAGTTCCTCCTTCT-3′ were annealed to form a dsDNA fragment, which was ligated with *Xba*I and *Eco*RI-digested pGL4.13EcoRI to produce the pGL4-LMNA plasmid. The identity of all constructs was confirmed by DNA sequencing.

### 2.3. Cell Culture and Transfection

A HEK293 line stably expressing FLAG-hAgo2 was established by using the Flp-In Expression System (Invitrogen) with the pcDNA5FLAG-hAgo2 plasmid and Flp-In-293 Cell Line (Invitrogen) according to the manufacturer's protocol. HeLa cells and HEK293-derivative cells were maintained in DMEM with 10% fetal bovine serum and antibiotics (100 U/mL penicillin and 100 *μ*g/mL streptomycin for both cell types, plus 100 *μ*g/mL Hygromycin B for HEK293-derivative cells). Synthetic small RNAs were transfected by using X-tremeGENE siRNA Transfection Reagent (Roche, Mannheim Germany). Reporter plasmids were transfected by using FuGENE 6 Transfection Reagent (Roche). HeLa cells were plated in 24-well plates (3 × 10^4^ cells/well) and in 12-well plates (6 × 10^4^ cells/well), and HEK293-derivative cells were plated in 10 cm dishes (2 × 10^6^ cells/dish) 24 h before transfection.

### 2.4. RNA Extraction and qRT-PCR Analysis

Total RNA was isolated from cultured cells with an RNeasy mini kit (QIAGEN, Hilden, Germany). To isolate RNA from immunoprecipitated hAgo2 protein, cells were lysed in 20 mM HEPES (pH 7.4), 150 mM NaCl, 1 mM MgCl_2_, 1 mM EGTA, 1 mM DTT, 1% EMPIGEN BB detergent (Sigma-Aldrich, St. Louis, MO, USA), 40 U/mL R Ribonuclease Inhibitor (Takara Bio Inc., Shiga, Japan) and 1% Protease Inhibitor Cocktail (Nacalai Tesque, Kyoto, Japan). FLAG-hAgo2 was immunoprecipitated with anti-FLAG M2 agarose beads (Sigma-Aldrich), and the beads were washed three times with lysis buffer and then directly suspended in the RNA extraction reagent ISOGEN (Nippon Gene Co., Ltd., Tokyo, Japan) to elute immunoprecipitated samples containing RNAs. RNA eluted in Isogen was isolated according to the manufacturer's protocol. 

 To analyze mRNA expression, a QuantiTect Reverse Transcription Kit (QIAGEN) and a Power SYBR Green PCR Master Mix (Applied Biosystems, Carlsbad, CA, USA) were used. To quantify the amount of antisense and sense strand in the RISC, a miScript PCR System (QIAGEN) was used. The primers for PCR analysis were 5′-GGAAGTCCAGTTCCTCCTTC-3′ for the LMNA antisense strand, 5′-GAAGGAGGAACTGGACTTCCA-3′ for the LMNA sense strand, 5′-GAAGGAGGCTGGACTTCCA-3′ for the LMNA sense strand with a deletion, and 5′-TAGCTTATCAGACTGATGTTG-3′ for miR-21; 5′-AGTCCATTCAGACATTGGGAG-3′ and 5′-GTTGTAGATGAAGGTGAGCAG-3′ for IRF9; and 5′-CAACCATGAGTACAAATGGTG-3′ and 5′-CTAGTAGGTTGTGTATTCCCA-3′ for IFIT1.

### 2.5. Immunoblotting and Reporter Analysis

For the immunoblotting assay, HeLa cells plated on 12-well plates were transfected with 5 pmol of RNA oligonucleotide. After 48 h, the cells were harvested by scraping them from culture dishes into hot 1× SDS sample buffer, and the lysates were separated by SDS-PAGE and analyzed by immunoblotting. The immunoblots were visualized and quantified by using an LAS-3000 imaging system (Fujifilm, Tokyo, Japan) and normalized to the levels of *β*-actin. Anti-Lamin A/C rabbit IgG (no. 2032) was purchased from Cell Signaling (Danvers, MA, USA). Anti-Lamin A rabbit IgG (L1293) and *β*-actin monoclonal mouse IgG (clone A-15) were from Sigma-Aldrich.

 For the reporter assay, HeLa cells plated in 24-well plates were transfected first with RNA oligonucleotide (0.25, 1.25, 2.5 pmol) 24 h before harvesting and then with reporter plasmid (100 ng/well reporter luciferase plasmid, pGL4-LMNA, and 25 ng/well renilla luciferase vector, pGL4.73; Promega) 30 min later. The luciferase activity was measured by using a dual-luciferase reporter assay system (Promega) with a Lumat LB9507 luminometer (Berthold Technologies, Bad Wildbad, Germany). As an internal control, renilla luciferase activity was used. The data reported represent the means and standard deviations of three independent experiments. 

### 2.6. Microarray Analysis

HeLa cells plated on 12-well plates were transfected with 5 pmol of RNA oligonucleotide, and RNA was extracted 48 h after transfection. 100 ng of total RNA was amplified and labeled by using an Ambion WT Expression Kit (Ambion) according to the supplier's protocol. HG-U133 Plus 2.0 arrays (Affymetrix, Santa Clara, CA, USA) were hybridized with 11 *μ*g of labeled cRNA, washed, stained, and scanned according to the protocol described in the Affymetrix GeneChip Expression Analysis Manual. Affymetrix data were extracted, normalized, and summarized with the robust multiaverage (RMA) method Expression Console.

## 3. Results

### 3.1. Design of Pre-miRNA-Based shRNAs

To assess the activity of synthetic shRNAs, we selected Lamin A/C (LMNA) as an RNAi target because this gene has been widely used as a positive control in siRNA experiments. Previous reports have developed miR-155-based vectors for RNAi [15, 16]; thus, we designed four shRNAs modeled on the pre-miRNA of human miR-155 (Figure 1(a)). All of the designed shRNAs had the same target site of the LMNA coding region but slightly different structures. The 17 nt loop structure and the 2 nt 3′ overhang were common parameters and are present in human pre-miR-155. It is known that Dicer efficiently cleaves dsRNA with a 2 nt 3′ overhang [11, 17] and that most pre-miRNAs have a 2 nt 3′ overhang generated by Drosha cleavage [18]. Two shRNAs (shRNA no. 1 and shRNA no. 1b) had a 24 bp stem length with or without an internal bulge, the other two (shRNA no. 2 and shRNA no. 2b) had a 21 bp stem length, generated by simply shortening the corresponding 24 bp stem shRNA at the loop side. Unlike in a previous report [11], the antisense strands were positioned 5′ to the loop. This position ensured that the antisense strand had a fixed 5′ end, irrespective of the position of cleavage by Dicer. The internal bulges in the stem region were introduced by a 2 nt deletion in the 3′-arm sequence mimic the structures of pre-miRNAs. These shRNAs were tested for their ability to silence the endogenous LMNA gene. HeLa cells were transfected with each shRNA, and the expression of LMNA protein was analyzed by immunoblotting. All four shRNAs knocked down expression of LMNA with similar efficiency (Figure 1(b)).

### 3.2. Potent RNAi Activity of Synthetic shRNA

**Figure 2:**
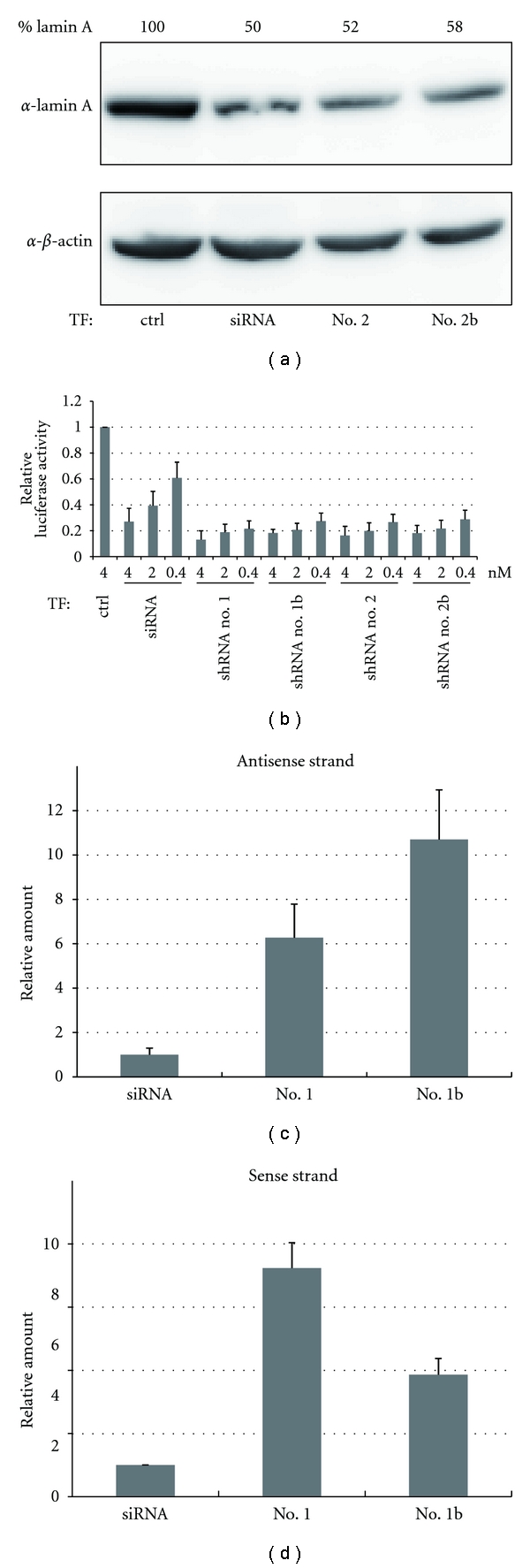
Comparison of the RNAi activity between synthetic shRNAs and their corresponding siRNAs. (a) The RNAi activity of siRNAs and shRNAs was analyzed by immunoblotting. (b) The RNAi activity was monitored by a reporter assay system. The luciferase activity ratio for the control siRNA (ctrl) was set as 1. (c), (d) The incorporation of transfected small RNA into the RISC was analyzed by qRT-PCR. Data were normalized against the amount of miR-21 in the RISC. The amount of siRNA incorporated was set as 1. Each graph point in (b), (c), and (d) represents the average (with s.d.) of three independent experiments.

Next, we compared the activity of siRNAs and shRNAs, by synthesizing siRNAs with same target sequence as the shRNAs. Immunoblotting analysis showed that shRNA no. 2 and shRNA no. 2b had RNAi activity comparable to that of the corresponding siRNA (Figure 2(a)). When shRNA no. 1 and shRNA no. 1b were used, essentially the same results were obtained (data not shown). 

 To analyze the activity more quantitatively, a more sensitive assay was conducted. We generated a luciferase construct that contained a perfectly complementary target site in its 3′-UTR and evaluated the reporter activity in cells cotransfected with various concentrations of synthetic small RNAs. All shRNAs, at all concentrations, were a 2- to 3-fold more potent RNAi trigger than the corresponding siRNA (Figure 2(b)). Importantly, even at the lowest concentration (0.4 nM), all shRNAs had silencing activity comparable to that observed at their 10-fold higher concentration (4 nM). These results are coincident with previous reports [11, 19] and indicate that synthetic shRNAs are more potent than their corresponding siRNAs. In this assay, we could not detect significant differences among the four shRNAs tested.

### 3.3. Efficiently Incorporation of Synthetic shRNAs into the RISC

As previously discussed [11], we reasoned that the more effective RNAi achieved by shRNAs as compared with siRNAs might reflect a difference in the efficiency of their incorporation into the RISC. We therefore examined the amount of antisense strands in the RISC generated from transfected small RNAs. Two shRNAs (no. 1 and no. 1b) were transfected into HEK293 cells stably expressing FLAG-tagged hAgo2 protein, which is a core component of the RISC [20]. Processed RNAs loaded into the RISC were isolated by coimmunoprecipitation with FLAG-tagged hAgo2 protein. As expected, real-time quantitative RT-PCR analysis revealed that the antisense strands derived from shRNAs were more efficiently incorporated into the RISC as compared with those derived from the corresponding siRNA (Figure 2(c)). Presumably, this efficient incorporation into the RISC underlies the highly potent silencing activity of shRNAs. 

 Interestingly, introducing an internal bulge in the stem increased the amount of antisense strands (compare no. 1b with no. 1 in Figure 2(c)). This might indicate that the equilibrium between the sense and antisense strands shifted towards antisense loading. We tested this equilibrium shift by quantifying the amount of antisense strands in the RISC. Indeed, introducing an internal bulge changed the equilibrium toward antisense loading (Figure 2(d)).

### 3.4. Off-Target Effects and Interferon Responses Induced by Synthetic shRNAs in HeLa Cells

**Figure 3:**
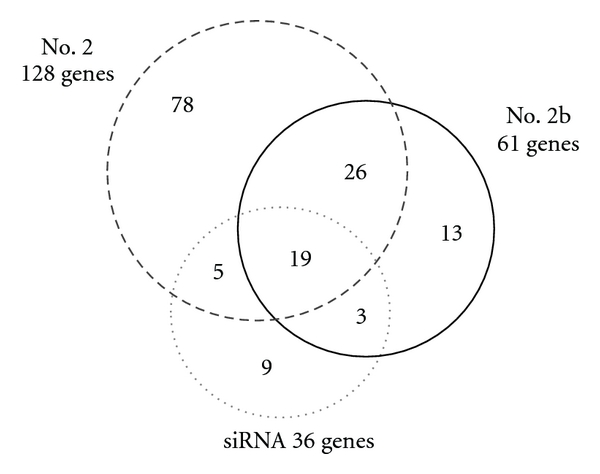
Off-target effects induced by synthetic shRNAs. HeLa cells were transfected with the indicated RNAs. After 48 h, the expression of about 20,000 protein-coding genes was analyzed by microarray analysis. The overlap of genes that showed a more than 2-fold decrease in expression as compared with the control siRNA-transfected sample is shown. The number in the overlapping region of the Venn diagram represents shared genes. Note that the LMNA gene is included in the commonly downregulated genes.

It is known that siRNAs can cause off-target effects [21]. Using microarray gene expression profiling, we compared the off-target effects of our synthetic small RNAs. From gene expression profiling, we identified genes that showed a more than 2-fold decrease in expression as compared with the control siRNA transfected sample. A Venn diagram of these downregulated genes is shown in Figure 3. As compared with siRNA, transfection of shRNAs caused a broader downregulation of nontargeted transcripts. This might be simply because of the efficient incorporation of synthetic shRNAs into the RISC and the consequent higher potent RNAi activity (Figure 2(c)). We noted that introducing a bulge decreased the number of off-target genes (compared no. 2b with no. 2). This phenomenon might reflect preferential incorporation of the antisense strand into the RISC, as occurred when shRNAs with a bulge were used (Figure 2(d)).

**Figure 4:**
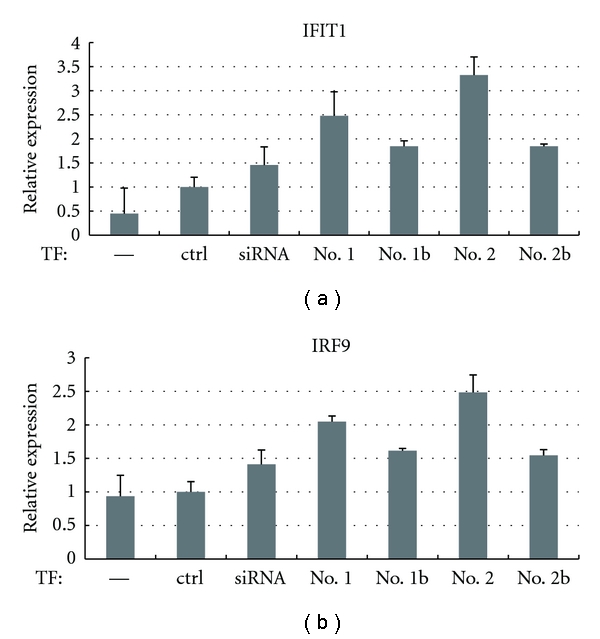
Interferon responses induced by synthetic shRNAs. HeLa cells were transfected with the indicated RNAs. After 48 h, the expression of two interferon-regulated genes IFIT1 and IRF9 was analyzed by qRT-PCR.

 Next, we examined the expression of interferon-related genes among the microarray data and found that IFIT1 [22] and IRF9 [23] were induced more than 2-fold by transfection of shRNA no. 2. We further analyzed the expression of these two genes by qRT-PCR analysis (Figure 4). Transfection of the siRNA upregulated IRF9 and IFIT1 expression by approximately 1.5-fold; however, transfection of shRNAs without a bulge caused slightly higher upregulation of these genes. Again, shRNAs with a bulge improved interferon responses. IFIT1 is known to be the mRNA that is most strongly induced in response to dsRNAs [22]. Introducing a bulge into the stem could circumvent this immune activation.

## 4. Discussion

In this study, we demonstrated that synthetic pre-miRNA-based shRNAs have more potent RNAi activity than their corresponding siRNA and found that their antisense strands are more efficiently incorporated into the RISC. A previous study also showed that synthetic shRNAs with a 27 nt stem and a 4 nt artificial loop have a significantly higher gene-silencing activity than conventional 21 nt siRNAs [11]. The authors demonstrated that these small RNAs are subjected to Dicer processing both *in vitro *and* in vivo* and speculated that siRNA duplexes generated by Dicer are efficiently loaded into the RISC. In a study using DNA vector-based shRNAs, two types of shRNA construct modeled on pre-miRNA and pri-miRNA transcripts were examined [24]. The results indicated that RNAi triggers that enter the RNAi pathway by a more natural route yield more effective silencing [25]. Here we demonstrated the efficient incorporation of shRNAs into the RISC, and our results are highly consistent with those of previous studies. Furthermore, introducing a bulge enhanced incorporation into the RISC and shifted the equilibrium toward antisense loading (Figures 2(c) and 2(d)). However, we did not observe a significant difference in RNAi activity among the synthetic shRNAs in our assay (Figure 2(b)), probably because they were all close to saturation.

 There are contradictory reports about the effects of stem length on RNAi activity. Some reports have shown that shRNAs with a 19 nt stem and a 9 or 10 nt loop have higher RNAi activity than shRNAs with longer stem [12, 26, 27]. This type of shRNA is widely used as a vector-based shRNA [28]. Interestingly, shRNAs with a 19 nt stem are not Dicer substrates; however, these shRNAs are thought to be incorporated into the RISC after bypassing Dicer processing [11]. Although we reasoned that the development of shRNAs based on naturally found structures is a promising approach, we should also examine and evaluate this type of shRNA.

 Unlike previously used shRNAs [11], our designed shRNAs have a shorter stem length (21 or 24 nt) and the loop derived from a pre-miRNA and are therefore expected to be less toxic. However, we observed that interferon responses were induced more strongly by shRNAs than by their corresponding siRNA. Interestingly, introducing a bulge also reduced interferon responses. These observations, albeit of a few examples, indicate that introducing a bulge has potential for improving the performance of shRNAs. Further examination of the position and structure of bulges is required. Chemical modifications of siRNAs have been extensively studied to increase stability, promote efficacy, minimize off-target effects, and reduce innate immune responses [5, 21]. Such modifications can be easily incorporated in synthetic shRNAs to improve siRNA stability and biological activity, similar to synthetic siRNAs. These are issues to be addressed in the future.
